# Elevated sodium leads to the increased expression of HSP60 and induces apoptosis in HUVECs

**DOI:** 10.1371/journal.pone.0179383

**Published:** 2017-06-12

**Authors:** Bojana Jakic, Maja Buszko, Giuseppe Cappellano, Georg Wick

**Affiliations:** Laboratory of Autoimmunity, Division of Experimental Pathophysiology and Immunology, Biocenter, Medical University of Innsbruck, Innsbruck, Austria; University of Palermo, ITALY

## Abstract

Atherosclerosis is the leading cause of death in the world. We have previously shown that expression of heat shock protein 60 (HSP60) on the surface of endothelial cells is the main cause of initiating the disease as it acts as a T cell auto-antigen and can be triggered by classical atherosclerosis risk factors, such as infection (e.g. *Chlamydia pneumoniae*), chemical stress (smoking, oxygen radicals, drugs), physical insult (heat, shear blood flow) and inflammation (inflammatory cytokines, lipopolysaccharide, oxidized low density lipoprotein, advanced glycation end products). In the present study, we show that increasing levels of sodium chloride can also induce an increase in intracellular and surface expression of HSP60 protein in human umbilical vein endothelial cells. In addition, we found that elevated sodium induces apoptosis.

## Introduction

Atherosclerosis is a multifactorial inflammatory condition of the arteries and the main disease-dependent cause of death worldwide [1]. It manifests itself clinically by an infiltration of mononuclear cells, i.e. T cells and macrophages into the arterial wall, of which the latter turn into foam cells due to accumulation of excess lipids. We have previously shown that T cells initiate the disease by recognizing and reacting towards endogenous HSP60 that, together with adhesion molecules, is expressed on the surface of endothelial cells when these are exposed to classical atherosclerosis risk factors [2]. T cells fail to ignore autologous HSP60 due to the fact that eukaryotic HSP60 and bacterial HSP65 are highly homologous proteins [3], leading to cross-recognition and as a result, to an autoimmune reaction both in atherosclerosis [4], and as reported in myasthenia gravis [5]. Moreover, it was shown in Hashimto thyroiditis that protein homology exists between HSP60 and thyroid molecules, and that HSP60 can be recognized by anti-thyroid antibodies [6]. More recently, an autoimmune role for HSP60 has also been implicated as a result of obesity in mice [7]. We have coined the term “The Autoimmune Concept of Atherosclerosis” for this, and it is now widely accepted [8–10].

Under physiological conditions, endothelial cells do not express HSP60 on their surface. However, when stressed by classical atherogenic risk factors, such as high blood pressure, obesity, diabetes, and cigarette smoking, HSP60 is expressed in the mitochondria and then transported to the cytoplasm and finally to the surface of endothelial cells at the predisposed atherosclerotic sites and thus acts as a “danger signal” for pre-existing HSP60 reactive T cells. We have previously reported that lipopolysaccharides (LPS) [11], *Chlamydia pneumoniae* [12], cigarette smoke extract (CSE) [13], pro-inflammatory cytokines, oxidized low-density lipoprotein [14], advanced glycation end products (AGE) [8], shear blood flow [15, 16] and heat shock [17, 18] all lead to the expression of HSP60 on the surface of endothelial cells together with adhesion molecules. Moreover, it has been shown that HSP60 derived peptides are also presented via both major histocompatibility (MHC) class I and II molecules [19–21]. On the other hand, our lab has shown that γδ T cells are present in the early stages of atherosclerosis at relatively high levels (10% of all T cells) [22]. This indicates that HSP60 recognition might also be possible in a non-MHC restricted fashion, since γδ T cells to not require MHC for activation [23].

The choice of lifestyle is a major determinant for the development of atherosclerotic disease, with intake of fat being a key player. Here we investigated the effects of a more recent factor that is in focus [24, 25], namely sodium chloride (NaCl, table salt), and its effect on HUVECs and their expression of HSP60. We have chosen HUVECs since they are accepted as a representative model of endothelial cells (ECs), easily accessible and the results are reliably extrapolatable to the *in vivo* situation [26].

Elevated plasma sodium is one of the main causes of increased blood pressure and subsequent chronic hypertension, eventually leading to atherosclerosis. There is extensive evidence that a reduction in dietary salt intake leads to a reduction in blood pressure [27, 28]. In direct relation to the immunopathology of atherosclerosis, it has been shown that elevated salt levels lead to the expression of adhesion molecules and chemo-attractants on HUVECs, more specifically VCAM-1 and E-selectin, both being important in the recruitment of mononuclear cells into the arterial wall [29]. In line with these results, and our previous results that VCAM-1, ICAM-1, E-selectin and HSP60 are simultaneously expressed on HUVECs when exposed to stress induced by atherogenic factors [14], we hypothesized that sodium chloride might also lead to an increase in HSP60 expression. Here, we report that increasing sodium chloride levels, from physiological to hypernatremic levels [30], leads to a directly increasing intracellular and surface expression of the HSP60 under static conditions in HUVECs. We also report an increase in apoptotic cells and a decrease in the number of cells, correlating to increased levels of sodium chloride. Jointly, these results indicate that elevated sodium chloride levels stress the endothelial cells and lead to an up-regulation of HSP60 and appearance of HSP60 on the surface. Endothelial cells thus become a direct target for autoreactive T cells, posing a risk for the development of atherosclerosis and other cardiovascular diseases.

## Material and methods

### Isolation and maintenance of primary HUVECs

Umbilical cords, collected with written informed patient consent were obtained from the Department of Gynecology and Obstetrics at the University Clinic of Innsbruck, and the study was conducted according to the guidelines of the Declaration of Helsinki and approved by the Ethics Committee of the Medical University of Innsbruck (Approval number UN2979 and UN4435). The cord was processed in RPMI 1640 (Lonza, Cat. No. 12–167) medium containing 1% penicillin/streptomycin (Lonza, Cat. No. 17-602E). The umbilical vein of the cord was flushed with cold RPMI 1640/Pen/Strep. Thereafter, the endothelial cells were detached with collagenase type IV (2.5mg/ml, Sigma, Cat. No. C5158) for 20 minutes at 37°C. The endothelial cells were then flushed out with endothelial cell basal medium (EBM) (Lonza, Cat. No. CC-3121) supplemented with Growth Factor SingleQuots (Lonza, CC-4133, containing bovine brain extract, ascorbic acid, human epidermal growth factor, fetal bovine serum, hydrocortisone and gentamicin/amphotericin-B). The cells were then centrifuged at 130 *g* for 5 minutes and plated on 0.2% gelatin-coated (Sigma, Cat. No. G1393) T75 flasks, for incubation at 37°C, 5% CO_2_, 95% humidity incubators and propagated in complete EBM (endothelial growth medium, EGM) medium. The medium was changed the following day and then every two days, until confluence was reached. The HUVECs were then briefly washed with sterile phosphate buffered saline (PBS) (pH 7.2), followed by 0.05% trypsin-EDTA (Gibco, Cat. No. 25300–064) or TrypLE (Gibco, Cat. No. 12604–013) treatment for 5 minutes at 37°C. The reaction was stopped with complete EGM medium, the cells were centrifuged and either re-plated, or frozen in cryoprotective freezing medium (Lonza, Cat. No. 12-132A) in the vapor phase of liquid nitrogen. All HUVECs that were used in this study were re-thawed passage 1–3 cells and used between passage 3 and 6. For re-thawing, the cells were added to fresh, warm complete EGM medium and maintained as described above. This study was conducted during 2016–2017.

### *In vitro* cell culture for immunofluorescence and flow cytometry

HUVECs were grown on 8-chamber slides (Sigma, Cat. No. C7057) for the immunofluorescence assays, and in 12-well plates for the flow cytometry assays, at an initial seeding concentration of 2,500cells/cm^2^. They were grown in complete EGM media (137 mM = physiological level) or complete EGM media supplemented with NaCl, devoid of iodine (CELLPURE 99.8%, Carl Roth, Cat. No. HN00.1) at concentrations of 145 mM, 158 mM, 173 mM, 188 mM. Medium was changed after 48 hours. In total, the cells were grown for 72 hours at which time point the cells grown in 137 mM had reached confluence.

### Immunofluorescence staining

The medium was aspired from the chambers and the cells were rinsed with sterile, warm (37°C) PBS. They were then fixed with either 1% paraformaldehyde (PFA) for 10 minutes at room temperature (RT) (for surface staining), or with 2% PFA for 10 minutes at RT followed by 100% methanol (MetOH) for 10 minutes at -20°C (membrane permeabilization for intracellular staining). Samples fixed with PFA+MetOH were then washed 3x5 minutes in 0.1%Triton-X/PBS at RT. Following this step, all slides were washed 3x5 minutes in PBS at RT. Samples were then blocked with 1% bovine serum albumin (BSA)-c/PBS (Aurion, Cat. No. 900.099) for 30 minutes at RT. After rinsing with PBS, the samples were incubated with the primary rabbit-anti-human HSP60 antibody (Santa Cruz Biotechnologies, Cat. No. sc-13966, clone H-300) together with a monoclonal mouse-anti-β-actin antibody (Sigma, Cat. No. A5441, clone AC-15) PBS for 30 minutes at RT. After washing with PBS 3x10 minutes, the samples were incubated with secondary donkey-anti-rabbit Alexa Fluor 568 (A568) (Abcam, Cat. No. ab175470) and donkey-anti-mouse A488 antibodies (Abcam, Cat. No. ab150105) in PBS for 30 min at RT. For negative controls, the primary antibodies were excluded or an isotype control was used (normal rabbit immunoglobulins fraction, Dako X0936). Samples were then washed 3x10 min in PBS at RT and the nuclei were then stained with Hoechst 33342 (Thermo Fisher). After washing in distilled water, the samples were mounted with Mowiol and coverslipped. All controls were negative (**S1 Fig**).

### Quantification of immunofluorescence

All images were acquired with a Zeiss AxioImager Microscope equipped with a MRm CCD camera. For quantification of HSP60 protein expression, exposure time was set at the sample incubated with the highest sodium concentration and kept unchanged for the acquisition of the same sample with decreasing sodium concentrations. The fluorescent images were then analyzed using FIJI software (Version 2.0.0-rc-49/1.51a) [31] by selecting one cell at a time in an image and measuring the area, integrated density and mean gray value. Using the calculation for corrected total cell fluorescence (CTCF) = integrated density–(area of selected cell × mean fluorescence of background readings), as described by McCloy et. al [32], the fluorescence intensity of each cell was calculated using Excel (Microsoft Office 2011 for Mac). For each image, three background areas were used to normalize against autofluorescence. Each biological sample was grown in five different conditions (i.e. different concentrations of sodium) and for each condition 5–10 images were acquired with a 40x objective, so that hundreds of cells per sample and per condition were available for analysis. This resulted in a more accurate mean fluorescence under each condition, which was then used for statistical analyses. Individual values are also provided in the Supporting information.

### Flow cytometry of surface HSP60 and CD31

HUVECs were grown in 12-well plates as described above. Thereafter, supernatant was aspired; the cells were washed with warm sterile PBS and de-attached with TrypLE. The reaction was stopped with complete EGM medium, the cells aspired and transferred into FACS tubes and centrifuged at 1200 rpm for 7 minutes at RT. Then, the cells were then incubated with Fc-blocking solution (anti-CD16/anti-CD32) (eBioscience, Cat. No. 14-0161-81C) for 10 minutes at +4°C. Thereafter, rabbit-anti-human HSP60 (Santa Cruz Biotechnologies, Cat. No. sc-13966, clone H-300) at 1:25 dilution together with a monoclonal mouse-anti-human CD31 FITC antibody (BD, Cat. No. 555445) at 1:20 dilution in 2%FCS/PBS was added and cells incubated for 30 minutes at +4°C. After washing with 2%FCS/PBS, the secondary donkey-anti-rabbit A568 (Abcam, Cat. No. ab175470) was added at 1:200 dilution, and incubated for 30 minutes at +4°C. After a final washing step, the cells were re-suspended in 2%FCS/PBS and acquired on a FACS Verse (BD Biosciences). The results were analyzed using FlowJo. As a negative control, secondary alone or mouse IgG1κ (BD, Cat. No. 51-35405X) were used.

### Quantification of cell numbers and apoptotic cells

Cells were quantified using flow cytometry and cell nuclei counting was carried out using FIJI software [31]. For flow cytometry, cells were grown as described above in 12-well plates. After 72 hours of incubation, the HUVECs were detached using 0.25 mM EDTA for 5–10 minutes at RT. Thereafter; the cells were washed in medium once and in PBS three times, followed by a final wash with Annexin V binding buffer (FITC labeled Annexin V Apoptosis Detection Kit, eBioscience, Cat. No. 88-8005-72) according to the manufacturer’s instructions. The cells were then stained with Annexin V FITC for 15 minutes at RT, followed by a wash with 3%FCS/PBS and finally acquired on a BD FACS Calibur (BD Biosciences) using the CellQuest software. All the cells from each sample were acquired. The data were then analyzed using FlowJo. Viability was checked by 7AAD exclusion (BD Biosciences Cat. No. 5168981E).

Cells were also counted using 5 images acquired by microscope with the 10x objective for each condition for each sample using FIJI software. The threshold was adjusted to a binary black and white image. The images were then converted to mask, which allows for the “watershed” function that separates adjacent cells into single units. Finally, the particles were analyzed, showing the outlines of each cell and the number of cells in that image. Cells on the edges were excluded.

### Statistical analyses

All statistical analyses and graphs were generated using GraphPad Prism (Version 5.0c). Outliers were excluded using Grubb´s test (http://graphpad.com/quickcalcs/grubbs1/). Linear relationships were analyzed using linear regression in GraphPad Prism. One-way ANOVA with Kruskal-Wallis non-parametric test and Dunn’s post-hoc test was used for multiple comparisons to the physiological level. Pearson two-tailed correlation analysis was used for testing correlation. *p*<0.05 was considered significant. The results are represented as mean ± SEM, unless otherwise indicated.

## Results

### Intracellular HSP60 protein expression in HUVECs increases with increasing sodium concentration

**Fig 1:**
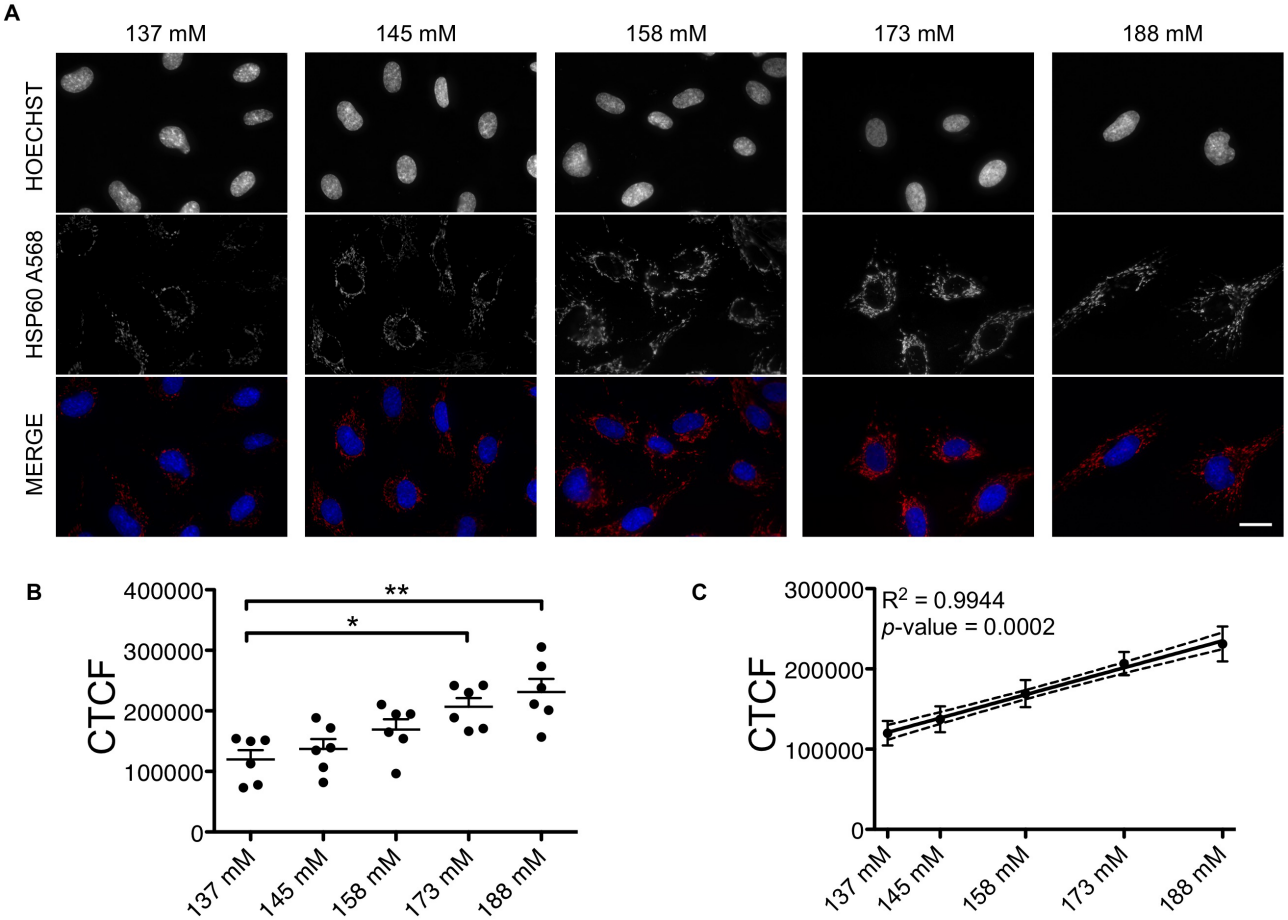
Intracellular HSP60 expression in HUVECs treated with increasing sodium concentrations. (A) Representative images of nuclei (Hoechst) and HSP60 (A568) staining under each experimental condition, on HUVECs fixed with 2% PFA + MetOH. Scale bar = 20 μm. (B) Quantification of HSP60 fluorescence staining intensity as explained in Material and Methods. Mean CTCF values ± SEM combined from two independent experiments. Each dot represents the CTCF readout from one donor (n = 6). (C) Linear regression of values presented in (B). Dotted lines show 95% confidence intervals. Mean ± SD. *p<0.05, **p<0.01. CTCF = corrected total cell fluorescence. mM = millimolar.

We cultured HUVECs in complete EGM media with sodium concentrations ranging from normal levels (137 and 145 mM) to hypernatriemic levels (>145 mM). After 72 hours, the cells in the lowest, physiological sodium condition had reached confluence, upon which the culturing was stopped for staining of HSP60. We found that HSP60 staining intensity increased with increasing sodium levels (**Fig 1A**). The staining was identical with the mitochondria location [33], indicating intracellular expression of HSP60.

As described in the Material and Methods section, quantitative analysis of the staining intensity showed that the 173 mM and 188 mM conditions resulted in significantly higher HSP60 intensities compared to the baseline 137 mM (**Fig 1B and S2 Fig**). More importantly, there was a highly significant (*p*-value = 0.0002, R^2^ = 0.9944) dependence of HSP60 expression on the concentration of sodium (**Fig 1C**).

### Surface HSP60 protein expression increases with increasing sodium concentration

**Fig 2:**
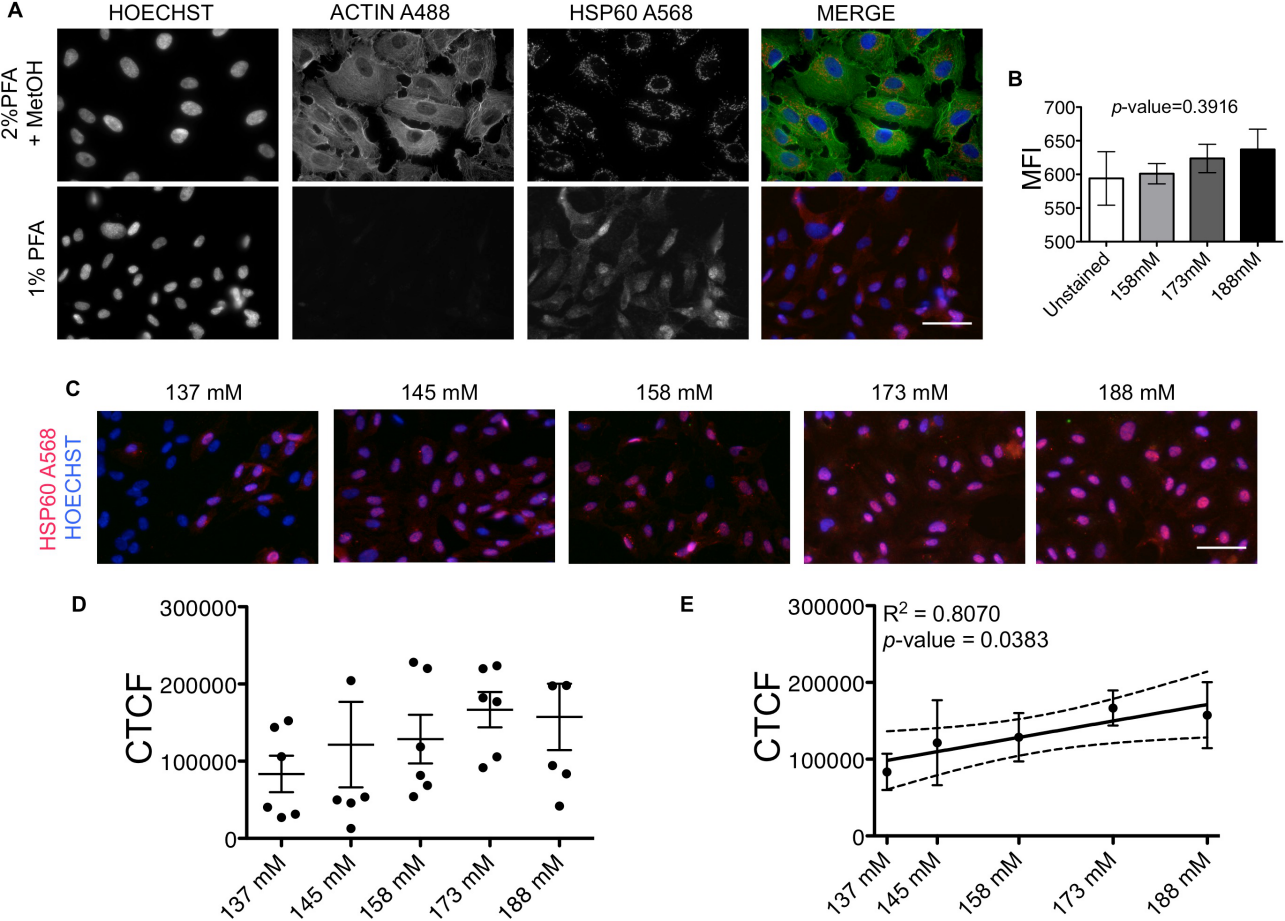
Surface staining of HSP60 on HUVECs treated with increasing sodium concentrations. (A) Representative images of nuclei (Hoechst), β-actin (A488) and HSP60 (A568) on HUVECs fixed with 2% PFA + MetOH or with 1% PFA. Scale bar = 50 μm. (B) MFI of surface HSP60 expression out of CD31^+^ endothelial cells. MFI of three donors ± SEM is shown. One-way ANOVA analysis was performed, p-value = 0.3916. (C) Representative images of surface staining of HSP60 under each experimental condition. Scale bar = 50 μm. (D) Quantification of surface HSP60 fluorescence staining intensity as explained in the Material and Methods. Mean CTCF values ± SEM combined from two independent experiments. Each dot represents the CTCF readout from one donor (n = 6). (E) Linear regression of values presented in (D). Dotted lines show the 95% confidence interval. Mean ± SD. CTCF = corrected total cell fluorescence. mM = millimolar. MFI = median fluorescence intensity.

As mentioned above, others and we have previously shown that HSP60 can be translocated to the surface of endothelial cells and act as a T cell auto-antigen [2, 4, 34, 35]. The display of HSP60 on the surface of endothelial cells is a key element in the “Autoimmune Concept of Atherosclerosis”. Therefore, we performed experiments aimed at investigating if increasing sodium levels in the HUVECs could lead to the translocation of HSP60 to the cell surface. For this purpose, we used co-staining for HSP60 and β-actin, the latter functioning as an internal positive control for intracellular staining and *vice versa* as an internal negative control for surface staining of HSP60. We found that plasma membrane permeabilization by fixation with 2% PFA + MetOH leads to intracellular staining of HSP60 where also β-actin is positively stained (**Fig 2A**, upper panel). Fixation with 1% PFA preserved plasma membrane integrity and led to surface staining of HSP60, with β-actin remaining unstained, confirming a surface detection of HSP60 (**Fig 2A**, lower panel). Moreover, we also assessed the co-staining of HSP60 with CD31, an endothelial surface marker [36], using flow cytometry (Fig 2B and S3 Fig). We could indeed confirm the immunohistochemical data, as the median fluorescence intensity (MFI) for HSP60 was increased with higher salt concentrations on CD31^+^ HUVECs, though not statistically significant, possibly because the HSP60 antibody is not optimal for flow cytometric staining. Assessment of surface staining of HSP60, showed that the intensity increased with the sodium levels (**Fig 2C**). Quantitative analysis of the signal showed a trend of higher levels of HSP60 in hypernatriemic conditions, although this was not statistically significant (**Fig 2D**). The association of sodium levels and surface HSP60 protein levels proved to be significant (*p*-value = 0.0383, R^2^ = 0.8070) as shown by regression analysis (**Fig 2E**).

### Elevated sodium levels induce apoptosis in HUVECs

**Fig 3:**
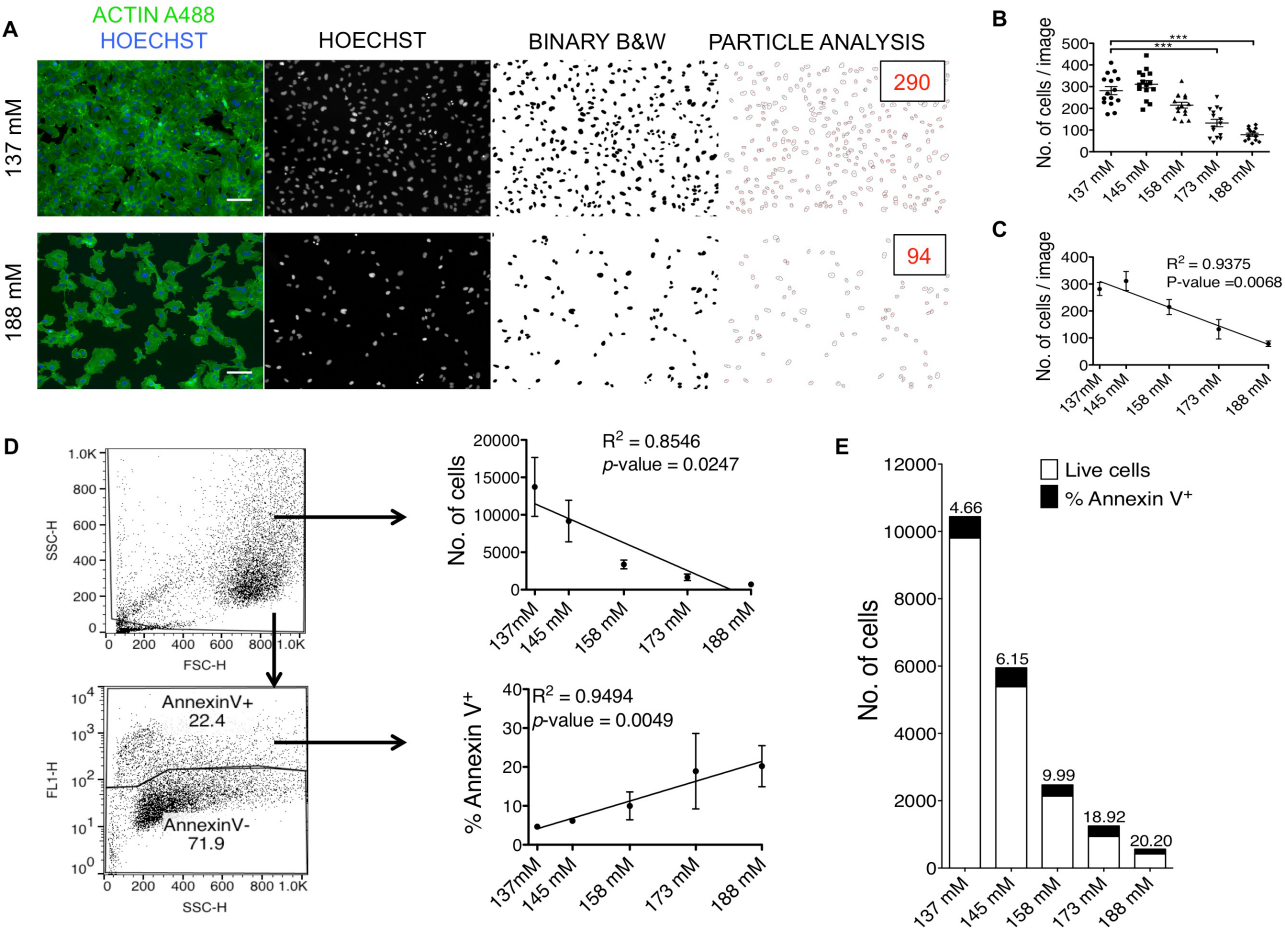
Quantification of total cells and apoptotic cells in HUVECs treated with increasing sodium concentration. (A) Representative images of HUVECs treated with 137 mM and 188 mM sodium, fixed with 2% PFA + MetOH and stained with Hoechst and anti-β-actin A488, and their subsequent enumeration using FIJI, as explained in Material and Methods. The number of cells per image is displayed in red under particle analysis. Scale bar = 100 μm. (B) Scatter plot of enumeration from acquired images of HUVECs treated with increasing sodium concentrations. Each dot represents number of cells per image. Mean ± SEM (C) Linear regression of values shown in (B). Mean ± SD (D) Representative flow cytometric gating strategies for enumeration of cells and Annexin V^+^ cells (apoptotic cells). Linear regression of FACS results for number of cells and percentage of apoptotic cells. Mean ± SD (E) Summary of flow cytometric enumeration of live and apoptotic cells (Annexin V^+^). The percentage of apoptotic cells within the total number of cells is shown on top of the bars. Data are from one experiment (n = 3). ***p<0.001. CTCF = corrected total cell fluorescence. mM = millimolar. B&W = black and white. No. = number.

To investigate if high sodium levels might affect cell numbers, we quantified the numbers of cells per acquired image, as explained in the Material and Methods and shown in **Fig 3A**. There were significantly fewer cells under hypernatriemic conditions (173 mM and 188 mM) compared to the baseline 137 mM (**Fig 3B**). This decrease was directly related to the increasing sodium concentrations as shown by regression analysis (*p*-value = 0.0068, R^2^ = 0.9375) (**Fig 3C**). To confirm these findings, cells were also counted by flow cytometry. The decrease in cell numbers was directly related to increasing sodium concentrations (*p*-value 0.0247, R^2^ = 0.8546) (**Fig 3D**, upper panel). However, the viability in the wells was constant in terms of percentage, as shown by 7AAD exclusion (**S2 Fig**). Furthermore, the staining with Annexin V showed that elevated levels of sodium lead to an increase in apoptosis (*p*-value = 0.0049, R^2^ = 0.9494) (**Fig 3D**, lower panel, as summarized in **Fig 3E**).

### Intracellular and surface HSP60 expression correlate with apoptosis

**Fig 4:**
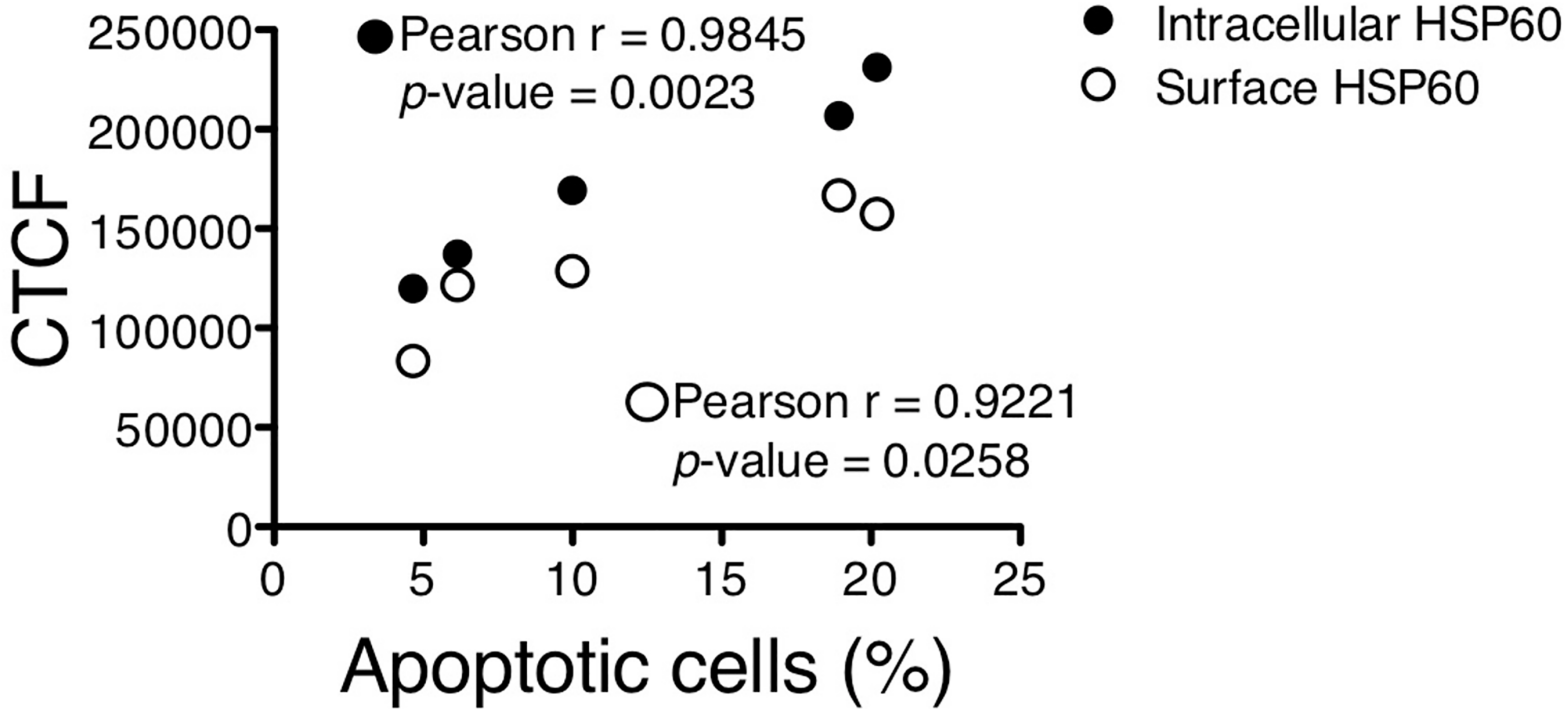
Correlation between HSP60 expression and apoptosis in HUVECS. Pearson correlation analysis. shows a significant correlation of intracellular and surface expression of HSP60 with the number of apoptotic cells, as determined by flow cytometry. Each dot represents the mean CTCF and corresponding percentage of apoptosis (Annexin V^+^) of 6 samples. CTCF = corrected total cell fluorescence.

Lastly, we found that the increase in intracellular and surface HSP60 and apoptosis, as a result of increase in sodium, were strongly correlated (r = 0.9845, *p*-value = 0.0023 for intracellular HSP60 *vs*. apoptosis, r = 0.9221, *p*-value = 0.0258 for surface HSP60 *vs*. apoptosis) (**Fig 4**).

## Discussion

Our hypothesis that increasing sodium concentrations above physiological levels have stress potential and lead to the directly correlated increase in intracellular and, more importantly, surface HSP60 protein localization was confirmed by our experimental observations. In addition, we found that the increase in sodium and HSP60 is correlated with an increase in apoptotic cells. The ability of sodium to induce endothelial cells to express HSP60 on the surface makes dietary salt an additional initiating risk factor for atherosclerosis, supporting our “Autoimmune Concept of Atherosclerosis”.

That high salt consumption is a risk factor for cardiovascular diseases is well known. Therefore, the scientific community and the World Health Organization (WHO) recommend lowering salt intake from 9–12 g/day to 5–6 g/day (corresponding to 2000 mg sodium) [37, 38]. In addition, individuals with already established hypertension can also display “salt sensitivity”—blood pressure varies in direct relation to dietary salt intake [39]. However, even in a healthy individual, the plasma levels of sodium can fluctuate during the day as a result of sudden high salt intake or due to dehydration. Physiological levels lie within the range of 134–148 mmol/l (mM), levels above 160 mmol/l are considered pathological and can be life-threatening [30, 40].

The exact mechanism by which sodium leads to pathologies of the vascular system is not yet elucidated in detail. Under normal physiological conditions, sodium levels are maintained by osmoregulation, by the renal renin-angiotensin-aldosterone system [41], the heart-derived atrial natriuretic peptide (ANP) [42], and by endothelial glycocalyx and sodium channels on vascular ECs [43, 44]. The endothelial glycocalyx consists of glycoproteins and proteoglycans that protrude from the ECs into the lumen as a thick mesh-like network. It functions as a primary physical barrier to protect ECs from harmful agents in the blood circulation [44]. Excess salt has been shown to be stored within certain tissues, such as the skin [45]. In the vascular endothelium, sodium channels are upregulated and this leads to influx of sodium into the cell and subsequent stiffening of the arteries. During stressful or inflammatory conditions, the glycocalyx layer is shed, enabling more sodium to enter the ECs. This is already the case with cultured HUVECs, as it has been shown that they have considerably less glycocalyx compared to ECs *in vivo* [46]. This is in line with our data showing that HSP60 is expressed intracellularly and on the surface even under the low sodium condition (137 mM) that is usually considered to be non-pathogenic.

It is known that many plant species and soil microorganisms up-regulate HSPs as a result of high salt content in the soil [47, 48]. Moreover, the expression of HSPs has been shown to be associated with hypertension. Specifically, circulating HSP60 and HSP70 have been found to correlate with the occurrence of hypertension [49, 50]. HSP60 is encoded in the nucleus and expressed in the mitochondria, but can also be found throughout the cellular cytoplasm [51, 52]. It is a chaperonin that is responsible for refolding of damaged proteins and transporting them into the mitochondria. It is generally considered to be a cell-protective protein [33]. However, we have repeatedly shown that, under stressful conditions, it becomes pathogenic [2, 4, 11–14, 17, 18]. We, and others, have also demonstrated that injection of ApoE^-/-^ or Ldlr^-/-^ mice (atheroprone knock-out mouse models) with HSP60 or the surrogate mycobacterial HSP65 leads to severe atherosclerosis [53–55]. Furthermore, we believe that salt-induced surface expression of HSP60 together with VCAM-1 and E-selectin [Dmitrieva and Burg 2015] renders ECs a target for pre-existing HSP60-auto-reactive T cells, firstly by attachment to the adhesion molecules and secondly by recognition of the autoantigen (HSP60). This still remains to be proven with for example *in vivo* studies using mice fed a diet with enriched sodium content.

HSPs are generally considered to be protective of apoptosis [56, 57]. They have been considered to be candidates for cancer therapies. Thus, accumulation of HSP60 in tumor cells has been reported, leading to resistance against cell death [51, 58]. On the other hand, it was demonstrated that accumulated HSP60 in the cytosol due to mitochondrial release in stressed cells activates pro-caspase-3, and hence induces apoptosis [59]. However, the same and other studies have also demonstrated that HSP60 that is present in the cytosol without apparent mitochondrial release has anti-apoptotic function by binding pro-caspase-3 [59, 60]. Therefore, the role of HSPs in apoptosis seems to be varied [61]. In our experimental setting, we found that an increase in sodium leads to a direct correlation between HSP60 expression and apoptosis. This can be explained by the “heat shock paradox”, according to which HSPs are protective upon initial cell insult, leading to healing of the cells. In contrast, if the cell has already been exposed to stress and injury beforehand, the subsequent heat shock response is detrimental and leads to apoptosis [57, 62]. Another term for this is hormesis, used in toxicology to explain that some agents are beneficial at low dose, but can be harmful at a high dose [63]. In our study, the HUVECs were already pre-stressed just by being put into cell culture, which manifests itself by a decrease in protective glycocalyx [46]. In the *in vivo* setting, ECs in pre-disposed areas are constantly insulted by turbulent blood flow, high fat content and smoking, so that HSP60 expression on the surface is in itself a pathological consequence. In addition, under atherogenic conditions, ECs are severely dysfunctional with drastically changed metabolism [64], with harmful insults emerging from both the blood flow and intima [65]. In atherosclerosis, ECs have also been shown to undergo apoptosis and to be sloughed off from the endothelial lining, leaving the plaque in a vulnerable state [66, 67].

Physiologically HSP is located on the mitochondrial membrane where it aids in protein folding and transport [52, 59]. In this study, we used a polyclonal rabbit antibody directed against eukaryotic HSP60. We have previously also shown HSP60 on the surface using the monoclonal antibody II-13 [68], originally developed by Singh and Gupta [69], which recognizes the amino acids 288–366, that are highly conserved and located in the center region of the protein. Others have shown HSP60 on the surface of Daudi cells, using antibodies towards both the N and C terminal portions of the protein [70]. Collectively, these results indicate that the intact HSP60 protein appears on the plasma membrane. Groundbreaking work has been done by other groups, which have shown that both the exosomal pathway [71] and the Golgi/endoplasmic reticulum pathway are involved in HSP60 deposition on the surface [72]. Within exosomes, HSP60 appeared to be monoubiquitinated [71]. However, the exact mechanism of HSP60 translocation, whether or not surface HSP60 is modified and with which other proteins it interacts, still remains to be elucidated.

Though excess of salt intake is a risk factor for vascular diseases, one should not, however, immediately jump to the conclusion that salt is harmful under all circumstances. In fact, a recent study showed that also a low intake in salt leads to an increased risk of cardiovascular events in both hypertensive and normotensive populations [73]. This is explained by an increase in renin and aldosterone levels, which, in the long run, has adverse effects on the lipid profile [74]. These authors do not recommend limiting sodium intake [73, 74] to the same extent as recommended by the WHO [38], but instead advise striving for a range that is close to the average intake in most countries today. This was 3.95 g sodium/day in 2010 [75], for the normotensive population. However, this report has been met with criticism from the scientific and medical community, as discussed in the Correspondence section of The Lancet (Volume 388, published 29 October 2016).

In conclusion, sodium chloride is an additional stressor and thus a risk factor for increased endothelial HSP60 expression and its subsequent translocation to the cell surface, rendering salt-induced HSP60 expression on ECs a direct target for pre-existing auto-reactive T cells. In addition, sodium-induced HSP60 expression is not protective but rather leads to apoptosis.

## Supporting information

S1 Fig Representative images of negative control staining for HSP60. (A) Shows 2%PFA+MetOH fixed HUVECs that were incubated with normal rabbit immunoglobulins fraction (Dako, X0936) and Donkey-anti-rabbit A568 (Abcam, ab175470) to the left and with rabbit-anti-HSP60 (Santa cruz, sc-18966) and donkey-anti-rabbit A568 (Abcam, ab175470) to the right. Orginal magnification 100X. (B) Shows 2%PFA+MetOH fixed HUVECs that were incubated with Donkey-anti-rabbit A568 (Abcam, ab175470 alone to the left and with rabbit-anti-HSP60 (Santa cruz, sc-18966) and donkey-anti-rabbit A568 (Abcam, ab175470) to the right. Original magnification 400X.
(PDF)

S2 Fig CTCF measurements. (A) Shows intracellular HSP60 and (B) shows surface HSP60 of CTCF values. Each dot represents one cells, red bars show mean ± SD. For each condition, 5–10 acquired images were analyzed. The mean of each condition was then used as the read-out.
(PDF)

S3 Fig Representative examples of CD31 FITC and HSP60 A568 staining using flow cytometry. (A) HUVECs are gated on SSC and FSC (left dot plot). Out of this gate, majority of the cells are expressing CD31 (middle dot plot), whereas staining with an isotype control did not result in a positive signal (right dot plot). X axis shows FSC and Y axis shows either SSC or FITC channel. (B) Out of CD31+ cells, HSP60 expression was quantified as MFI. Here is shown a representative of an unstained control with secondary alone and a sample that was incubated with 188mM salt concentration. X axis shows fluorescence intensity and Y axis shows normalized cell counts. SSC = side scatter, FSC = forward scatter, MFI = median fluorescence intensity.
(PDF)

S4 Fig Percentage of live cells as analyzed by 7AAD exclusion using flow cytometry. Mean ± SEM (n = 3).
(PDF)
