# Supplementary material for: Elevated sodium leads to the increased expression of HSP60 and induces apoptosis in HUVECs
Source: PLoS One. 2017 Jun 12;12(6):e0179383. doi: 10.1371/journal.pone.0179383 (PMC5467851; doi:10.1371/journal.pone.0179383)
Supplement: S1 Fig — (A) Shows 2%PFA+MetOH fixed HUVECs that were incubated with normal rabbit immunoglobulins fraction (Dako, X0936) and Donkey-anti-rabbit A568 (Abcam, ab175470) to the left and with rabbit-anti-HSP60 (Santa cruz, sc-18966) and donkey-anti-rabbit A568 (Abcam, ab175470) to the right. Orginal magnification 100X. (B) Shows 2%PFA+MetOH fixed HUVECs that were incubated with Donkey-anti-rabbit A568 (Abcam, ab175470 alone to the left and with rabbit-anti-HSP60 (Santa cruz, sc-18966) and donkey-anti-rabbit A568 (Abcam, ab175470) to the right. Original magnification 400X. (PDF) [file pone.0179383.s001.pdf]

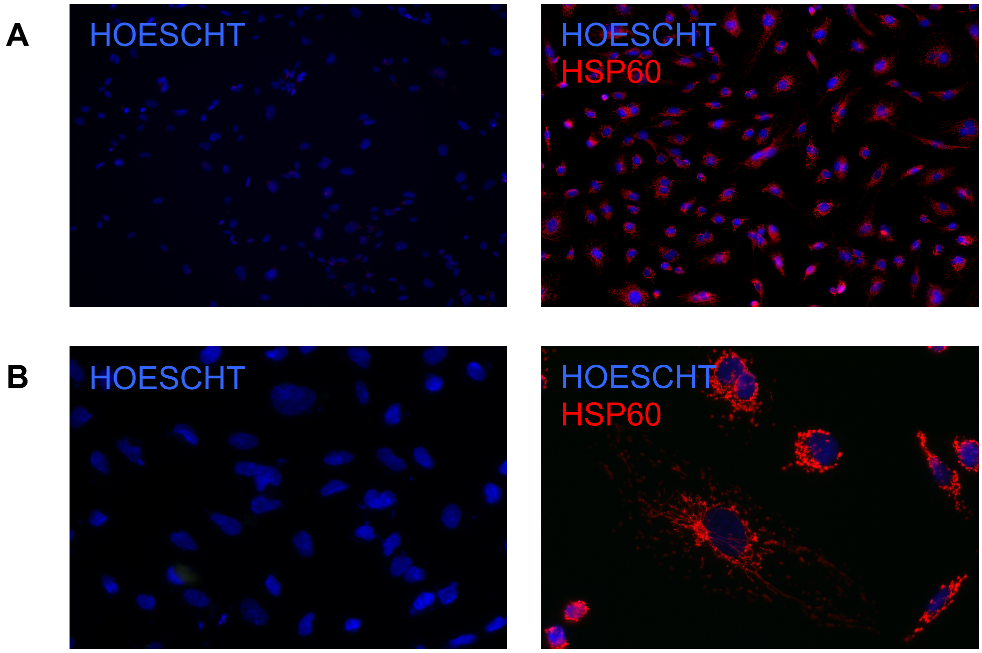

S1 Fig. Representative images of negative control staining for HSP60.
(A) Shows 2%PFA+MetOH fixed HUVECs that were incubated with normal rabbit immunoglobulins fraction (Dako, X0936) and Donkey-anti-rabbit A568 (Abcam, ab175470) to the left and with rabbit-anti-HSP60 (Santa cruz, sc-18966) and donkey-anti-rabbit A568 (Abcam, ab175470) to the right. Orginal magnification 100X. (B) Shows 2%PFA+MetOH fixed HUVECs that were incubated with Donkey-anti-rabbit A568 (Abcam, ab175470 alone to the left and with rabbit-anti-HSP60 (Santa cruz, sc-18966) and donkey-anti-rabbit A568 (Abcam, ab175470) to the right. Original magnification 400X
