# Supplementary material for: Elevated sodium leads to the increased expression of HSP60 and induces apoptosis in HUVECs
Source: PLoS One. 2017 Jun 12;12(6):e0179383. doi: 10.1371/journal.pone.0179383 (PMC5467851; doi:10.1371/journal.pone.0179383)
Supplement: S2 Fig — (A) Shows intracellular HSP60 and (B) shows surface HSP60 of CTCF values. Each dot represents one cells, red bars show mean ± SD. For each condition, 5–10 acquired images were analyzed. The mean of each condition was then used as the read-out. (PDF) [file pone.0179383.s002.pdf]

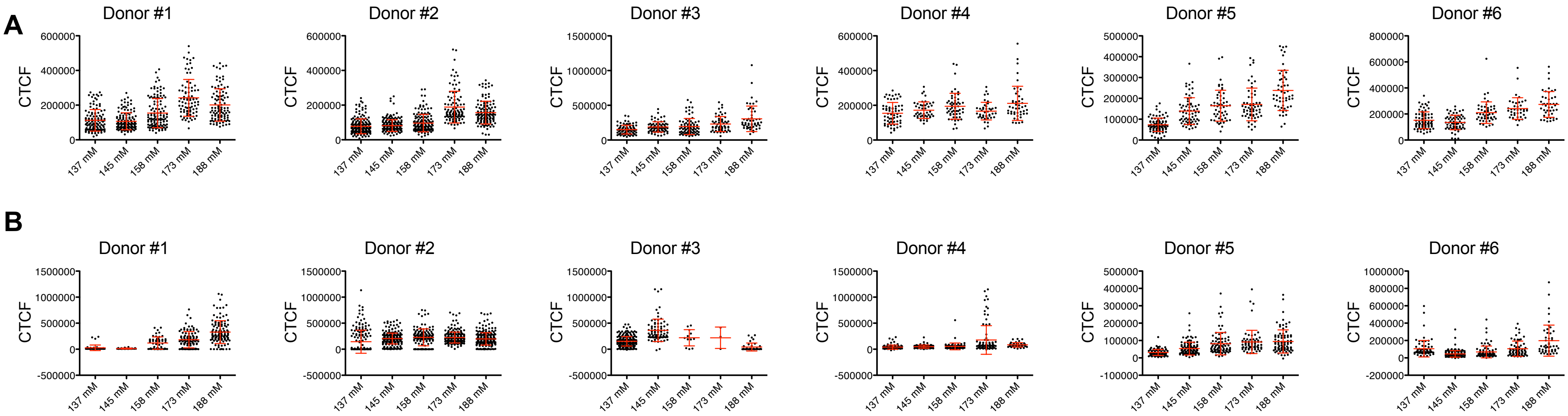


**S2 Fig. CTCF measurements**. (A) Shows intracellular HSP60 and (B) shows surface HSP60 of CTCF values. Each dot represents one cells, red bars show mean ± SD. For each condition, 5-10 acquired images were analyzed. The mean of each condition was then used as the read-out
