# Supplementary material for: Elevated sodium leads to the increased expression of HSP60 and induces apoptosis in HUVECs
Source: PLoS One. 2017 Jun 12;12(6):e0179383. doi: 10.1371/journal.pone.0179383 (PMC5467851; doi:10.1371/journal.pone.0179383)
Supplement: S3 Fig — (A) HUVECs are gated on SSC and FSC (left dot plot). Out of this gate, majority of the cells are expressing CD31 (middle dot plot), whereas staining with an isotype control did not result in a positive signal (right dot plot). X axis shows FSC and Y axis shows either SSC or FITC channel. (B) Out of CD31+ cells, HSP60 expression was quantified as MFI. Here is shown a representative of an unstained control with secondary alone and a sample that was incubated with 188mM salt concentration. X axis shows fluorescence intensity and Y axis shows normalized cell counts. SSC = side scatter, FSC = forward scatter, MFI = median fluorescence intensity. (PDF) [file pone.0179383.s003.pdf]

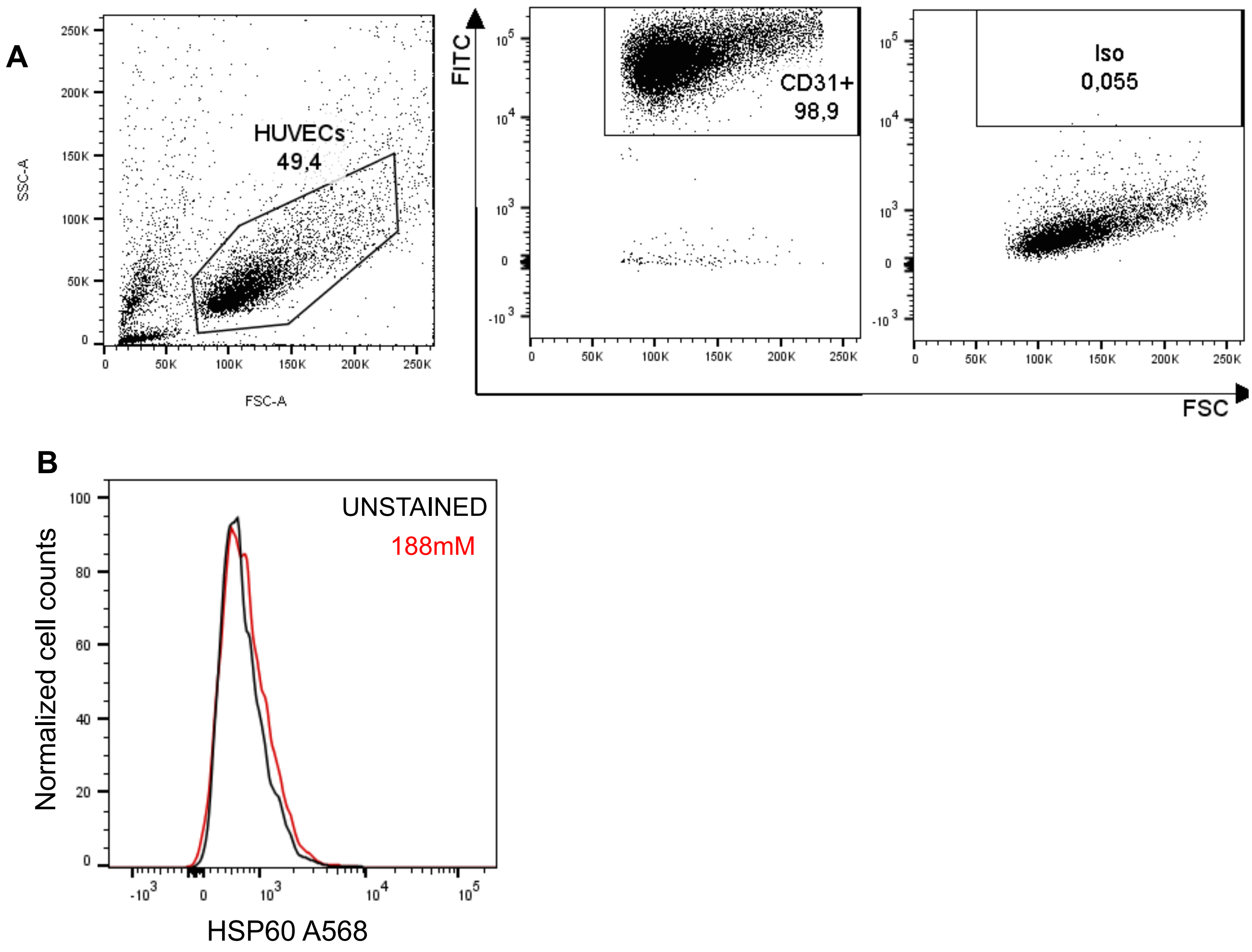

S3 Fig. Representative examples of CD31 FITC and HSP60 A568 staining using flow cytometry.

(A) HUVECs are gated on SSC and FSC (left dot plot). Out of this gate, majority of the cells are expressing CD31 (middle dot plot), whereas staining with an isotype control did not result in a positive signal (right dot plot). X axis shows FSC and Y axis shows either SSC or FITC channel. (B) Out of $CD31^+$ cells, HSP60 expression was quantified as MFI. Here is shown a representative of an unstained control with secondary alone and a sample that was incubated with 188mM salt concentration. X axis shows fluorescence intensity and Y axis shows normalized cell counts. SSC = side scatter, FSC = forward scatter, MFI = median fluorescence intensity
