# Supplementary figures and images for: Elevated sodium leads to the increased expression of HSP60 and induces apoptosis in HUVECs
Source: PLoS One. 2017 Jun 12;12(6):e0179383. doi: 10.1371/journal.pone.0179383 (PMC5467851; doi:10.1371/journal.pone.0179383)

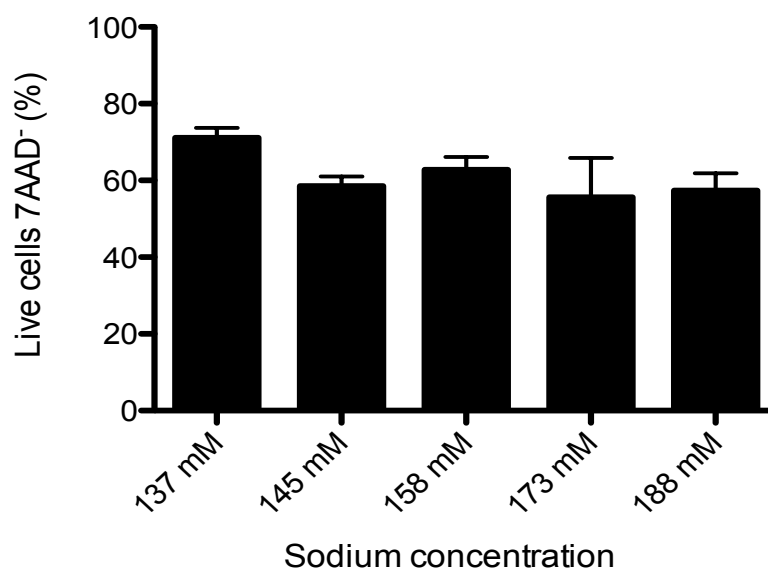

**S4 Fig. Percentage of live cells as analyzed by 7AAD exclusion using flow cytometry.**  
Mean  $\pm$  SEM (n=3)

Supplement: S4 Fig — Mean ± SEM (n = 3). (PDF) [file pone.0179383.s004.pdf]
